# When should a diagnosis of influenza be considered in adults requiring intensive care unit admission? Results of population-based active surveillance in Toronto

**DOI:** 10.1186/cc10331

**Published:** 2011-07-28

**Authors:** Stefan P Kuster, Kevin C Katz, Joanne Blair, James Downey, Steven J Drews, Sandy Finkelstein, Rob Fowler, Karen Green, Jonathan Gubbay, Kazi Hassan, Stephen E Lapinsky, Tony Mazzulli, Donna McRitchie, Janos Pataki, Agron Plevneshi, Jeff Powis, David Rose, Alicia Sarabia, Carmine Simone, Andrew Simor, Allison McGeer

**Affiliations:** 1 Mount Sinai Hospital, 600 University Avenue, Toronto, ON, M5G 1X5, Canada; 2 University of Toronto, 1 King's College Circle, Toronto, ON, M5S 1A8, Canada; 3 North York General Hospital, 4001 Leslie Street, Toronto, ON, M2K 1E1, Canada; 4 Ontario Agency for Health Protection and Promotion, 81 Resources Road, Toronto, ON, M9P 3T1, Canada; 5 Toronto East General Hospital, 825 Coxwell Avenue, Toronto, ON, M4C 3E7, Canada; 6 ProvLab Calgary, University of Calgary, 3030 Hospital Drive NW, Calgary, AB, T2N 4W4, Canada; 7 The Scarborough Hospital, 3030 Birchmount Road, Toronto, ON, M1W 3W3, Canada; 8 Sunnybrook Health Sciences Centre, 2075 Bayview Avenue, Toronto, ON, M4N 3M5, Canada; 9 Credit Valley Hospital, 2200 Eglinton Avenue West Mississauga, Mississauga, ON, L5M 2N1, Canada

## Abstract

**Introduction:**

There is a paucity of data about the clinical characteristics that help identify patients at high risk of influenza infection upon ICU admission. We aimed to identify predictors of influenza infection in patients admitted to ICUs during the 2007/2008 and 2008/2009 influenza seasons and the second wave of the 2009 H1N1 influenza pandemic as well as to identify populations with increased likelihood of seasonal and pandemic 2009 influenza (pH1N1) infection.

**Methods:**

Six Toronto acute care hospitals participated in active surveillance for laboratory-confirmed influenza requiring ICU admission during periods of influenza activity from 2007 to 2009. Nasopharyngeal swabs were obtained from patients who presented to our hospitals with acute respiratory or cardiac illness or febrile illness without a clear nonrespiratory aetiology. Predictors of influenza were assessed by multivariable logistic regression analysis and the likelihood of influenza in different populations was calculated.

**Results:**

In 5,482 patients, 126 (2.3%) were found to have influenza. Admission temperature ≥38°C (odds ratio (OR) 4.7 for pH1N1, 2.3 for seasonal influenza) and admission diagnosis of pneumonia or respiratory infection (OR 7.3 for pH1N1, 4.2 for seasonal influenza) were independent predictors for influenza. During the peak weeks of influenza seasons, 17% of afebrile patients and 27% of febrile patients with pneumonia or respiratory infection had influenza. During the second wave of the 2009 pandemic, 26% of afebrile patients and 70% of febrile patients with pneumonia or respiratory infection had influenza.

**Conclusions:**

The findings of our study may assist clinicians in decision making regarding optimal management of adult patients admitted to ICUs during future influenza seasons. Influenza testing, empiric antiviral therapy and empiric infection control precautions should be considered in those patients who are admitted during influenza season with a diagnosis of pneumonia or respiratory infection and are either febrile or admitted during weeks of peak influenza activity.

## Introduction

The 2009 H1N1 influenza pandemic had a substantial effect on ICUs [1] in that pandemic 2009 influenza (pH1N1) infection was associated with severe hypoxemia, multisystem organ failure, requirements for prolonged mechanical ventilation and the need for rescue therapies [2-5].

Many observational cohort studies, both from the 2009 pandemic and of seasonal influenza pre-pandemic, have found that antiviral therapy for influenza is associated with significantly improved outcomes, particularly when it is initiated within 48 hours of the onset of symptoms [6-8]. Optimal management of severe influenza thus depends on the ability to recognize those patients admitted to the ICU who require empiric therapy for influenza pending the results of diagnostic testing. However, data about clinical characteristics that help to identify patients at high risk of influenza infection upon hospital or ICU admission during influenza season are sparse [9,10]. The aim of this study was to identify populations of patients with increased probabilities of influenza infection among subjects admitted to ICUs during the 2007/2008 and 2008/2009 influenza seasons as well as the second wave of the 2009 H1N1 influenza pandemic.

## Materials and methods

### Setting and manoeuvre

The Toronto Invasive Bacterial Diseases Network (TIBDN) is a collaborative network of microbiology laboratories, infection control practitioners and public health departments that performs population-based surveillance for infectious diseases in south-central Ontario [11-13]. Six acute care hospitals from the TIBDN participated in active surveillance for laboratory-confirmed influenza requiring ICU admission during the 2007/2008 and 2008/2009 influenza seasons, and three of these hospitals performed active surveillance during the second wave of the pH1N1 influenza pandemic. All admissions to adult medical or medical/surgical ICUs were included.

Prior to the 2007/2008 influenza season, attending physicians agreed that, during influenza seasons, nasopharyngeal (NP) swabs were clinically indicated in patients requiring ICU admission who presented with any acute respiratory or cardiac illness (independent of body temperature) or in patients with any febrile illness without a clear, nonrespiratory aetiology. During each influenza season, study staff screened all admissions daily and suggested orders for NP swabs (if they had not already been ordered) from all patients with any acute cardiac or respiratory illness or any febrile illness without a clear nonrespiratory source. Demographic and medical information was collected from each patient by chart review. Fever upon ICU admission was defined as being present if the first body temperature measured after ICU admission was ≥38.0°C, and the diagnosis was defined as recorded in each chart. Respiratory symptoms were defined as any upper or lower respiratory symptoms such as coryza, cough, wheezing or shortness of breath. NP swabs were tested for the presence of influenza by PCR and viral culture at the Ontario Public Health Laboratory. Some specimens were also tested by direct fluorescent antigen detection (DFA) or enzyme immunoassay (EIA) at individual hospitals.

For the purposes of the study, influenza season was defined as starting when the proportion of positive influenza tests among specimens submitted to the Ontario Public Health Laboratory for viral testing was >5% for two consecutive weeks and as ending when the proportion of positive tests was <5%. The peak season was *a priori *defined as any week in which the proportion of submitted specimens yielding influenza was >15% [10].

### Statistical analyses

Data were double-entered, cleaned and analyzed using SAS version 9.1 software for PC (SAS Institute, Cary, NC, USA). Data were analyzed for all patients who were tested for influenza infection. Differences in medians were analyzed using the Wilcoxon rank-sum test or the Kruskal-Wallis test, and differences in group proportions were assessed using a χ^2 ^test or Fisher's exact test as appropriate. We performed multivariable logistic regression analyses to evaluate independent predictors of seasonal and pandemic H1N1 influenza. The variables 'age ≥65 years', 'temperature ≥38.0°C upon admission', 'admitting diagnosis respiratory infection', 'admitting diagnosis respiratory failure' and 'week with >15% specimens positive' were considered for inclusion in multivariable models based on clinical judgment and previously published literature [10] in a manner that minimized the Akaike Information Criterion, with final models representing those that best balanced parsimony and fit [14]. The Hosmer-Lemeshow Goodness-of-Fit Test was used to assess model fit. The limited number of outcomes was factored in when building the models to prevent overfitting. Likelihoods were calculated as binomial proportions with 95% confidence intervals. Two-sided *P *values < 0.05 were considered statistically significant.

### Ethics approval

The study was approved by the Research Ethics Boards of all participating hospitals. Written informed consent was obtained as required from all participants or their authorized representatives.

## Results

### Influenza seasons

**Figure 1:**
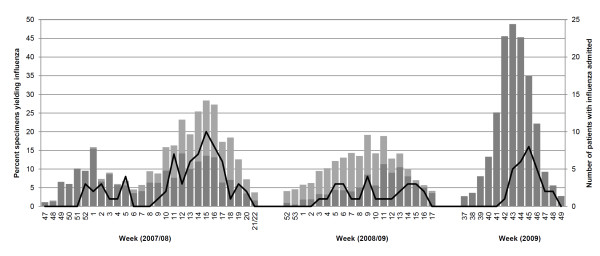
**Comparison of influenza activity by laboratory surveillance in Ontario**. Data are expressed as the percentage of specimens submitted to reference virology laboratories yielding influenza, 2007/08 and 2008/09 influenza seasons and second wave of the 2009 H1N1 influenza pandemic (light grey bars: influenza B; dark grey bars: influenza A), as well as the corresponding number of patients admitted to participating ICUs with influenza (black line).

The influenza season in Toronto in 2007/2008 was bimodal, with a first season beginning on 16 December 2007 (week 51) and ending on 2 February 2008 (week 5) and a second season beginning on 24 February 2008 (week 9) and ending on 17 May 2008 (week 20) (Figure 1) [15]. Influenza activity was predominantly influenza A(H1N1) during the first season and mixed influenza A(H3N2) and influenza B during the second season. The 2008/2009 influenza season began on 18 January 2009 (week 3) and ended on 25 April 2009 (week 16), with influenza B, influenza A(H1N1) and influenza A(H3N2) circulating [16]. The second wave of the 2009 H1N1 pandemic started on 11 October 2009 (week 41) end ended on 5 December 2009 (week 48). Influenza activity was almost exclusively pH1N1 [17].

### Results of surveillance

**Figure 2:**
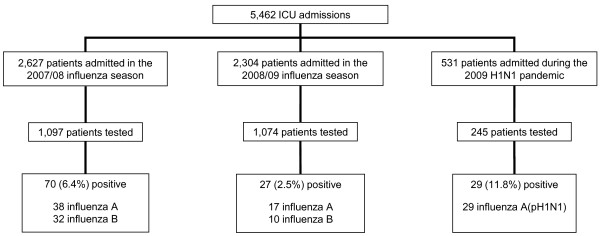
**Study subjects**. Flowchart of study subjects requiring admission to ICUs in Toronto during the 2007/08 and 2008/09 influenza seasons and the second wave of the 2009 H1N1 influenza pandemic.

During the 2007/2008 and 2008/2009 influenza seasons and the second wave of the 2009 H1N1 pandemic, a total of 5,462 patients were admitted to participating ICUs. Among these patients, 2,416 patients were tested for influenza and 2,360 (97.7%) met the eligibility criteria for testing (Figure 2). Compliance with testing differed among the three influenza seasons (90.4% of eligible patients tested during the 2009 H1N1 influenza pandemic, 89.4% during the 2008/2009 influenza season and 63.0% during the 2007/2008 influenza season; *P *< 0.001) and among the six study hospitals (proportion of eligible patients tested ranging from 63.9% to 90.4%; *P *< 0.001). Specimens from eligible patients were more likely to be submitted if the patient was febrile (80.2% vs. 74.6%; *P *= 0.035) or reported respiratory symptoms upon admission (78.0% vs. 73.3%; *P *= 0.006), if the patient was >65 years of age (76.7% vs. 72.4%; *P *= 0.007), if admission did not occur during peak influenza weeks (77.6% vs. 71.8%; *P *< 0.001) and if the admission diagnosis was 'pneumonia', 'other respiratory infection', 'asthma exacerbation', 'chronic obstructive pulmonary disease (COPD) exacerbation' or 'respiratory failure' (82.7% vs. 72.9%; *P *< 0.001).

A total of 126 patients (5.2% of those tested) were identified as being infected with influenza. During the 2007/2008 influenza season, 54.3% (38 of 70) of isolates obtained were influenza A (Figure 2). Seven influenza A isolates were subtyped: two (28.6%) were influenza A(H1N1) and five (71.4%) were influenza A(H3N2). Similarly, 63.0% (17 of 27) of isolates obtained during the 2008/2009 influenza season were influenza A. Fifteen of these were subtyped: three (20.0%) were influenza A(H1N1) and twelve (80.0%) were influenza A(H3N2). All 29 influenza isolates identified during the 2009 pandemic were subtyped and confirmed to be pH1N1.

DFA tests were positive for influenza in 2.6% (13 of 492) of tests submitted, EIAs were positive for influenza in 1.4% (18 of 1,255), viral cultures were positive for influenza in 2.8% (39 of 1,381) and PCRs were positive for influenza in 4.9% (110 of 2,263). Among patients with at least one positive test for influenza, DFA results were positive in 68.4% (13 of 19), EIA results were positive in 29.0% (18 of 62), viral culture results were positive in 50.6% (39 of 77) and PCR results were positive in 97.3% (110 of 113).

### Patient characteristics

The characteristics of patients with influenza admitted during the 2007/2008 and 2008/2009 influenza seasons and the second wave of the 2009 influenza pandemic are shown in Tables 1 and 2. Compared to the 2007/2008 and 2008/2009 influenza seasons, patients requiring ICU admission due to pH1N1 were more likely to be <65 years of age (23 of 29 (79.3%) vs. 30 of 97 (30.9%); *P *< 0.001) and more likely to be admitted with a diagnosis of 'pneumonia' or 'other respiratory infection' (17 of 29 (58.6%) vs. 34 of 97 (35.1%); *P *= 0.023). The proportion of males, febrile patients, patients presenting with respiratory symptoms, patients admitted from long-term care facilities and patients admitted with other diagnoses among patients with influenza infection did not differ between the 2007/2008 and 2008/2009 influenza seasons and the 2009 influenza pandemic. Analyses according to influenza subtype (seasonal influenza A vs. influenza B vs. pH1N1) revealed statistically significant differences with regard to patient age and admission temperature (Table 2). Analyses according to seasonal influenza A subtypes (influenza A(H1N1) vs. influenza A(H3N2)) could not be performed due to the low numbers of subtyped isolates.

**Table 1:** Characteristics of patients admitted to ICUs^a^

Characteristics	Influenza-positive/total, *n *(%)	
	
	2007/2008 and 2008/2009 influenza seasons	2009 H1N1 pandemic, second wave
Total number of patients	97/2,171 (4.5)	29/245 (11.8)
Gender		
Males	50/1,197 (4.2)	19/127 (15.0)
Females	46/972 (4.7)	10/118 (8.5)
Age, years		
All patients, median (IQR)	72.0 (59.4 to 80.5)	67.5 (52.8 to 79.7)
18 to 44	6/168 (3.6)	9/35 (25.7)
45 to 64	24/596 (4.0)	14/78 (18.0)
65 to 84	54/1,147 (4.7)	6/107 (5.6)
≥85	13/260 (5.0)	0/25 (0.0)
Admitted from long-term care facility	7/95 (7.4)	4/13 (30.8)
Temperature on admission, °C		
All patients, years, median (IQR)	36.6 (36.0 to 37.2)	36.7 (36.2 to 37.5)
<38.0°C	74/1,964 (3.8)	18/217 (8.3)
≥38.0°C	23/207 (11.1)	11/28 (39.3)
Respiratory symptoms upon hospital admission		
Absent	9/628 (1.4)	1/55 (1.8)
Present	87/1,449 (6.0)	27/184 (14.7)
Admission diagnosis		
Respiratory infection^b^	34/298 (11.4)	17/49 (34.7)
Respiratory failure^c^	22/212 (10.4)	3/25 (12.0)
Sepsis/fever	4/101 (4.0)	2/15 (13.3)
Any cardiac diagnosis	26/939 (2.8)	3/52 (5.8)

^a^IQR: interquartile range; ^b^'pneumonia' and 'other respiratory infection'; ^c^'chronic obstructive pulmonary disease exacerbation', 'asthma exacerbation' and 'respiratory failure'.

**Table 2:** Characteristics of influenza-positive patients admitted to ICUs^a^

Characteristics	Influenza A (seasonal) (*n *= 55)	Influenza B (*n *= 42)	Pandemic H1N1 (*n *= 29)	*P *value
Male gender, *n *(%)	26 (47.3)	24 (58.5)	19 (65.5)	0.24
Median age, years (IQR)	77.6 (63.7 to 83.6)	73.3 (57.2 to 80.8)	50.1 (43.6 to 61.9)	<0.001
18 to 44, *n *(%)	4 (7.3)	2 (4.8)	9 (31.0)	0.001
45 to 64, *n *(%)	10 (18.2)	14 (33.3)	14 (48.3)	0.015
65 to 84, *n *(%)	34 (61.8)	20 (47.6)	6 (20.7)	0.002
≥85, *n *(%)	7 (12.7)	6 (14.3)	0 (0.0)	0.09
Median admission temperature, °C (IQR)	37.1 (36.4 to 37.7)	36.7 (36.1 to 37.8)	37.7 (37.3 to 38.2)	0.004
>38.0°C, *n *(%)	13 (23.6)	10 (23.8)	11 (37.9)	0.32
Respiratory symptoms, *n *(%)	51 (92.7)	36 (87.8)	27 (96.4)	0.44
Admission diagnosis				
Respiratory infection^b^	18 (32.7)	16 (38.0)	17 (58.6)	0.06
Respiratory failure^c^	11 (20.0)	11 (26.2)	3 (10.3)	0.26
Sepsis	3 (5.5)	1 (2.4)	2 (6.9)	0.65
Cardiac	16 (29.1)	10 (23.8)	3 (10.3)	0.15

^a^IQR: interquartile range; ^b^'pneumonia' and 'other respiratory infection'; ^c^'chronic obstructive pulmonary disease exacerbation', 'asthma exacerbation' and 'respiratory failure'.

### Predictors of seasonal and pandemic (H1N1) 2009 influenza infection in patients admitted to ICUs

In multivariable analysis, body temperature ≥38.0°C and an admission diagnosis of 'pneumonia/other respiratory infection' were independently associated with both seasonal and pH1N1 influenza (Table 3). An admission diagnosis of 'COPD exacerbation/asthma exacerbation/respiratory failure' independently predicted seasonal influenza but not pH1N1 infection. Of note, the lack of predictive ability of this variable for pH1N1 was independent of patient age. Age <65 years was independently associated with pandemic (H1N1) 2009 influenza but not with seasonal influenza.

**Table 3:** Predictors of influenza infection in adult patients admitted to ICUs^a^

	Univariable analysis		Multivariable analysis^b^	
	
Predictor	Odds ratio (95% CI)	*P *value	Odds ratio (95% CI)	*P *value
Seasonal influenza A and B (2007/2008 and 2008/2009)				
Age ≥65 years	1.2 (0.8 to 1.9)	0.37	1.2 (0.8 to 1.9)	0.37
Female gender	1.1 (0.8 to 1.7)	0.53	-	-
Temperature ≥38.0°C upon admission	3.2 (2.0 to 5.2)	<0.001	2.3 (1.4 to 3.9)	0.002
Respiratory symptoms upon admission	4.4 (2.2 to 8.8)	<0.001	-	-
Admission diagnosis				
Respiratory infection^c^	3.7 (2.4 to 5.7)	<0.001	4.2 (1.6 to 3.7)	<0.001
Respiratory failure^d^	2.9 (1.8 to 4.8)	<0.001	4.2 (2.6 to 6.9)	<0.001
Week with >15% positive specimens	2.6 (1.7 to 3.9)	<0.001	2.5 (1.6 to 3.7)	<0.001
Pandemic 2009 H1N1 influenza				
Age ≥65 years	0.2 (0.1 to 0.5)	<0.001	0.5 (0.3 to 0.8)	0.004
Female gender	0.5 (0.2 to 1.2)	0.12	-	-
Temperature ≥38.0°C upon admission	7.2 (2.9 to 17.6)	<0.001	4.7 (1.7 to 13.6)	0.004
Respiratory symptoms upon admission	9.3 (1.2 to 70.0)	0.009	-	-
Admission diagnosis			-	-
Respiratory infection^c^	8.1 (3.5 to 18.6)	<0.001	7.3 (3.0 to 18.1)	<0.001
Respiratory failure^d^	1.1 (0.3 to 3.8)	0.92	-	-
Week with >15% positive specimens	1.4 (0.5 to 4.2)	0.57	-	-

^a^95% CI: 95% confidence interval. ^b^Variables included in the final model for seasonal and pandemic 2009 H1N1 influenza, respectively, are those where odds ratios and *P *values are reported in the multivariable analysis. The Akaike Information Criterion values for the final model of seasonal and pandemic 2009 H1N1 influenza, respectively, were 721.4 and 140.0. ^c^'Pneumonia' and 'other respiratory infection'. ^d^'Chronic obstructive pulmonary disease exacerbation', 'asthma exacerbation' and 'respiratory failure'.

### Percentage of patients with seasonal and pandemic (H1N1) 2009 influenza in different patient populations

Table 4 depicts the percentage of patients with seasonal influenza and pH1N1 in various patient populations. The percentage of patients with seasonal influenza was elevated sixfold above baseline (baseline proportion of seasonal influenza during the 2007/2008 and 2008/2009 influenza seasons = 0.045) in febrile patients admitted with 'pneumonia', 'other respiratory infection', asthma exacerbation', 'COPD exacerbation' or 'respiratory failure' during weeks of peak influenza activity. In relation to baseline (baseline proportion of pH1N1 during the second wave of the 2009 influenza pandemic = 0.118), the percentage of patients with pH1N1 was elevated more than twofold in afebrile patients admitted with 'pneumonia' or 'other respiratory infection' and more than fivefold if these patients were admitted with a fever ≥38.0°C. However, a considerable fraction of patients with influenza were not in high-risk groups, particularly in the case of seasonal influenza. During influenza seasons, patients with 'pneumonia', 'other respiratory infection', asthma exacerbation', 'COPD exacerbation' or 'respiratory failure' admitted during peak weeks comprised only 39% of all patients with influenza. During the pandemic, patients with an admission diagnosis of 'pneumonia' or 'other respiratory infection' comprised 59% of all patients with influenza (Table 4).

**Table 4:** Proportion of subjects with influenza in different populations of screened patients requiring admission to ICUs^a^

Influenza season	Age	Admission diagnosis	Admission temperature	Timing during season	Proportion with influenza (95% CI)	Proportion of total influenza cases	Proportion of patients screened
2007/2008 and 2008/2009	Any	Respiratory infection^b ^or respiratory failure^c^	≥38°C	Peak weeks	0.27 (0.15 to 0.43)	0.124	0.020
			<38°C	Peak weeks	0.17 (0.11 to 0.24)	0.268	0.071
			≥38°C	Early or late weeks	0.12 (0.04 to 0.26)	0.052	0.019
			<38°C	Early or late weeks	0.05 (0.03 to 0.08)	0.134	0.124
2009/2010 H1N1 pandemic	Any	Respiratory infection^b^	≥38°C	Any time	0.70 (0.35 to 0.93)	0.241	0.041
			<38°C	Any time	0.26; 0.13 to 0.42)	0.345	0.159

^a^OR: odds ratio; 95% CI: 95% confidence interval; ^b^'pneumonia' and 'other respiratory infection'; ^c^includes 'chronic obstructive pulmonary disease exacerbation', 'asthma exacerbation' and 'respiratory failure'.

## Discussion

The findings of our study may assist clinicians in decision making regarding influenza testing and empiric antiviral treatment of adult patients admitted to ICUs during future influenza seasons. In prospective surveillance for laboratory-confirmed influenza infection in patients admitted to the ICUs of six hospitals in Toronto during the 2007/2008 and 2008/2009 influenza seasons and to the ICUs of three hospitals during the second wave of the 2009 H1N1 influenza pandemic, admission body temperature ≥38.0°C, admission diagnosis of 'pneumonia/other respiratory infection' or 'COPD/asthma exacerbation/respiratory failure' and admission during weeks of peak influenza activity were independent predictors for seasonal influenza. Overall, 27% of patients meeting all three criteria had laboratory-confirmed influenza infection. Age <65 years, admission body temperature ≥38°C and admission diagnosis of 'pneumonia/other respiratory infection' were predictive for pH1N1: 70% of patients with all three characteristics who were admitted during the second wave of the 2009 patient had pH1N1 infection.

An increasing number of studies suggest that specific antiviral therapy is effective in reducing the morbidity and mortality associated with influenza [18-21], and current guidelines for both seasonal and pandemic influenza recommend the treatment of patients requiring hospitalization for influenza [6,7,22]. However, it is also clear that antiviral therapy is more effective when started in the early stages of infection [22-28]. Thus, optimal management of patients dictates the use of empiric therapy, and understanding the pretest probability of infection is necessary for its rational use. The differences between the positive predictive value for influenza in patients in peak and off-peak influenza weeks during influenza seasons emphasize the need for intensivists to know whether influenza is circulating in their area and where they are in each "season" when deciding on testing and treatment for influenza. As a rule of thumb, influenza seasons last from 10 to 16 weeks, with peak weeks being the central 4 to 6 weeks. The 2007/2008 influenza season in Toronto was an exception. The onset of the season varies by year and by geographic region within countries and is now usually declared locally by public health units in North America.

The identification of patients shedding influenza virus is also important for infection prevention, especially in ICUs, where influenza outbreaks may be fatal [29,30]. Hospital influenza outbreaks are not uncommon [31], and expert opinion suggests that additional precautions for patients admitted with influenza are an important element of control of transmission of influenza in ICUs [22,32,33]. Our data support the implementation of additional precautions empirically until influenza is ruled out for all patients admitted with pneumonia or other respiratory infection if they are febrile or are admitted during weeks of peak influenza activity.

RT-PCR is the only diagnostic test with adequate performance characteristics for the diagnosis of influenza in adult patients requiring hospital admission [9,22]. As shown previously by other groups, we have confirmed that RT-PCR is more sensitive than viral culture or DFA for rendering an influenza diagnosis, and these tests are, in turn, more sensitive than commercially available rapid influenza tests (that is, EIA) [34-36].

There are a number of limitations to our study. First, sampling from three years in a single geographic area may limit the generalizability of our results. We minimized selection bias by applying broad inclusion criteria for testing: All patients who presented with acute respiratory or cardiac illness or with febrile illness without clear nonrespiratory aetiology were eligible for inclusion. However, only 75% of eligible patients were tested for influenza, suggesting that the proportion of patients actually infected might be higher than our estimate. Data collection by chart review limited the number of risk factors considered, including differences that might have been found between vaccinated and unvaccinated patients. In addition, rather than individual specific respiratory symptoms (for example, cough, shortness of breath), we assessed only respiratory symptoms overall. However, the factors we identified were selected to be easily available for all patients and have the value of simplicity. Finally, it has previously been shown that patients who are admitted to the hospital with influenza infection do not constitute a homogeneous group [37]. The proportion of patients who need specific antiviral therapy to control influenza infection is unknown, in contrast to those who are able to control viral replication but have complications as a result of influenza. There is a need for further study to define the potential of antiviral therapy and the causes of hospitalization due to influenza.

## Conclusions

Among those adult patients who are admitted to the ICU during influenza season with a diagnosis of pneumonia or respiratory infection and who are either febrile or admitted during weeks of peak influenza activity, the probability of influenza infection may be high enough to warrant consideration of influenza testing, empiric antiviral therapy and/or empiric infection control precautions. However, although our simple rules may result in improved identification of patients with influenza infection, a significant proportion of patients who present without these characteristics will still be missed and our understanding of which patients will benefit from treatment remains incomplete. These persisting difficulties highlight the need for further research to enable better identification of patients admitted with atypical presentations, to understand when patients with influenza are infectious and to clarify which patients will benefit from antiviral therapy.

## Key messages

• Optimal management of severe influenza depends on the ability to recognize those patients admitted to the ICU who require empiric therapy and additional precautions for influenza pending the results of diagnostic testing.

• Influenza testing, empiric antiviral therapy and empiric infection control precautions should be considered in the small proportion of patients admitted during influenza season with a diagnosis of pneumonia or respiratory infection and who are either febrile or admitted during weeks of peak influenza activity.

• Although identification of patients may be improved with the application of this simple rule, a significant proportion of patients with influenza infection will be missed. Further research is needed with regard to strategies for improved identification of influenza patients admitted with atypical presentations.

## Abbreviations

COPD: chronic obstructive pulmonary disease; DFA: direct fluorescent antigen detection; EIA: enzyme immunoassay; NP: nasopharyngeal; pH1N1: pandemic 2009 H1N1 influenza; RT-PCR: reverse transcriptase polymerase chain reaction; TIBDN: Toronto Invasive Bacterial Diseases Network.

## Competing interests

AM has investigator-initiated research studies funded by Hoffman-La Roche Ltd. and GlaxoSmithKline Ltd. AS is a member of the Speakers' Bureau for Hoffmann-La Roche Ltd. All other authors have no competing interests.

## Authors' contributions

SPK performed the statistical analysis and drafted the manuscript. KCK, JB, JD, SJD, SF, RF, KG, JG, KH, SEL, TM, DM, JP, AP, JP, DR, AS, CS and AS participated in the design of the study and contributed to the acquisition of data. AM conceived of and designed the study, participated in study coordination and helped to draft the manuscript. All authors read and approved the final manuscript.
